# Supplementary material for: LSH mediates gene repression through macroH2A deposition
Source: Nat Commun. 2020 Nov 6;11:5647. doi: 10.1038/s41467-020-19159-0 (PMC7648012; doi:10.1038/s41467-020-19159-0)
Supplement: Supplementary file 1 — Supplementary information [file 41467_2020_19159_MOESM1_ESM.pdf]

Supplementary information

LSH mediates gene repression through macroH2A deposition

Ni et al.

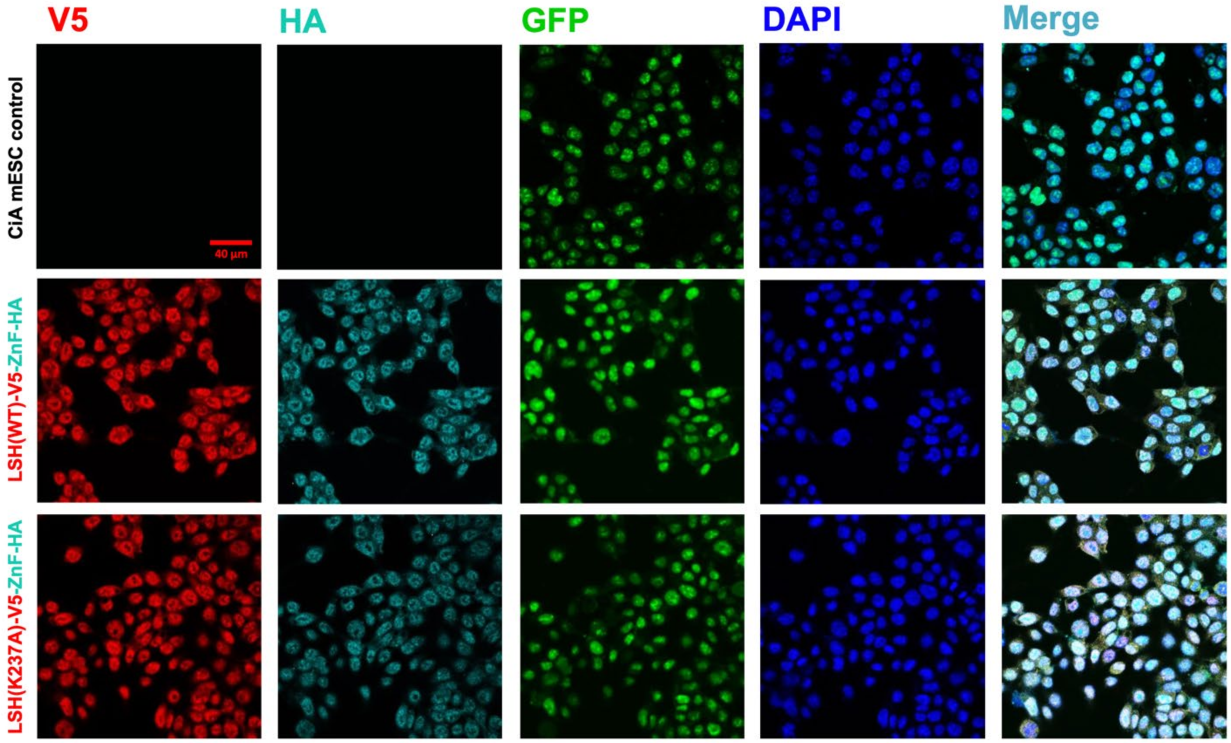

Supplementary Fig. 1: Detection of fusion proteins in *CiA: Oct4* mES cells. IF assay to show *CiA: Oct4* mES cells expressing LSH (WT) or ATP mutant LSH (K 237 A)-FRB-V5 protein and ZnF-FKBP-HA protein by staining with V5 (red) and HA antibodies (cyan) in conjunction with DAPI (blue) counterstaining. Cells without transfection were used as controls. Scale bar, 40 µm.

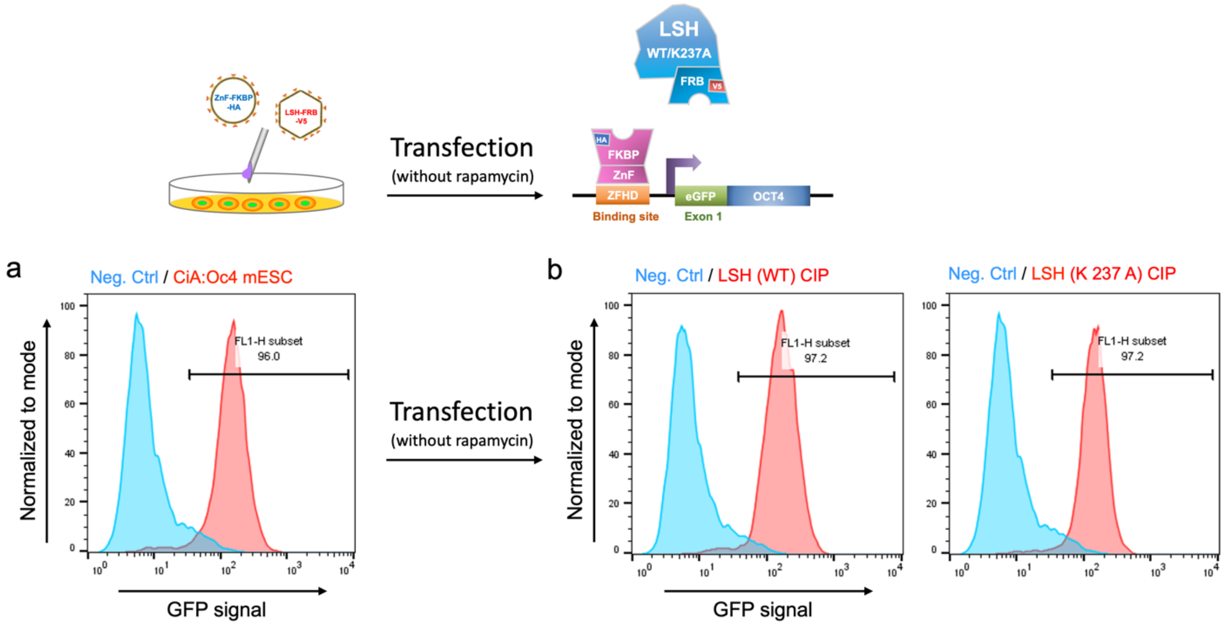

Supplementary Fig. 2: Fusion protein expression does not affect GFP expression in engineered mouse ES cells. a The gating strategy to separate GFP (+) cells from GFP (-) cells by high relative fluorescence flow cytometry analysis to display GFP-reporter expression level in engineered mouse ES cells with one modified *CiA: Oct4* allele compared to unmodified ES cells. **b** Comparison of GFP level between *CiA: Oct4* mouse ES cells expressing LSH (WT) or ATP mutant LSH (K 237 A)-FRB-V5 protein and ZnF-FKBP-HA protein without rapamycin treatment. Unmodified mouse ES cells lacking GFP expression served as negative controls (Neg. Ctrl).

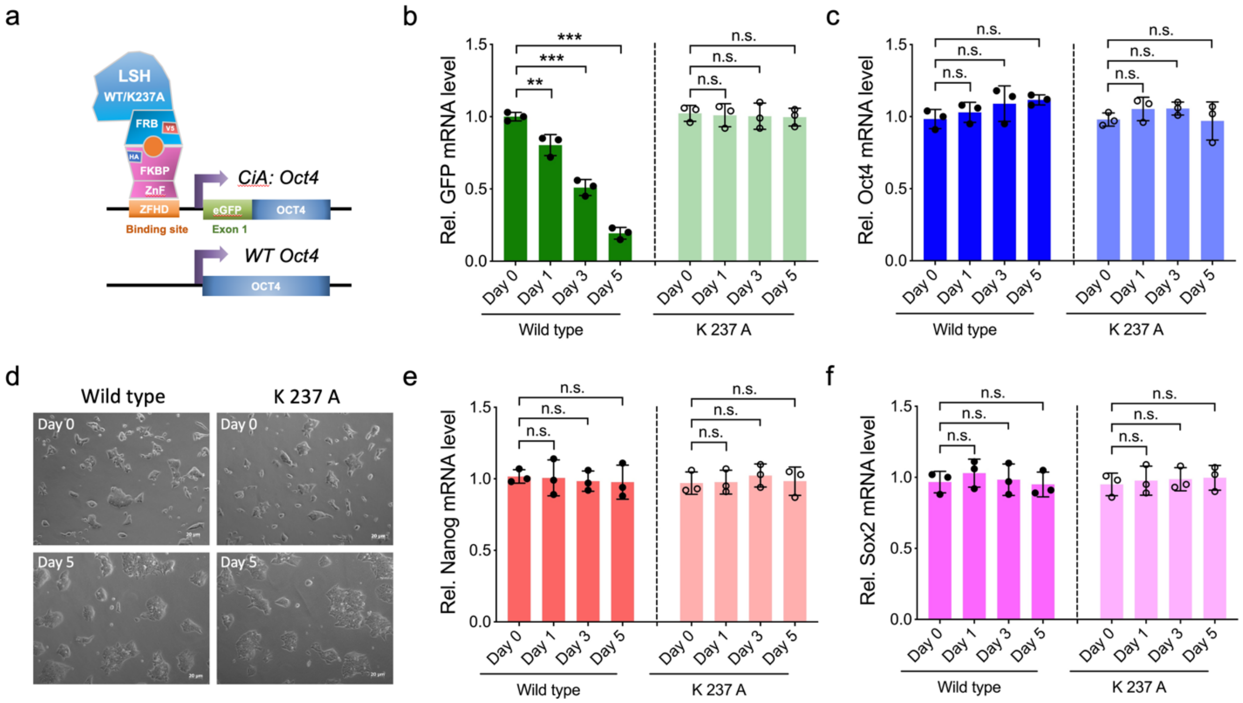

Supplementary Fig. 3: GFP repression does not affect *Oct4* mRNA generated by the unaltered allele. a Schematic representation of FRB-tagged wild type (WT) and ATP mutant LSH (K 237 A) specific recruitment to the engineered allele in the CIP system after by rapamycin treatment. **b-c** The amount of *GFP* (**b**) and *Oct4* (**c**) mRNA was measured by RT-qPCR in *CiA: Oct4* mES cells tethered with wild type or ATP mutant LSH (K 237 A) after rapamycin treatment at each time point. **adjusted p = 0.0074 and ***adjusted p < 0.0001. n.s. means not significant. **d** Cellular morphology of *CiA: Oct4* mES cells with recruitment of wild type or ATP mutant LSH (K 237 A) after 0 and 5 days of rapamycin treatment. Scale bar, 20 µm. **e-f** RT-qPCR analysis to determine the expression of other pluripotency genes, *Nanog* (**e**) and *Sox2* (**f**), mRNA levels in the wild type and ATP mutant LSH (K 237 A) CIP systems with rapamycin treatment at each time point. n.s. means not significant. Data are represented as mean ± SD. One-way ANOVA with Tukey's multiple comparison test (**b**, **c**, **e**, **f**). (**b**, **c**, **e**, **f**) representative of three independent experiments. Source data are provided as a Source Data file.

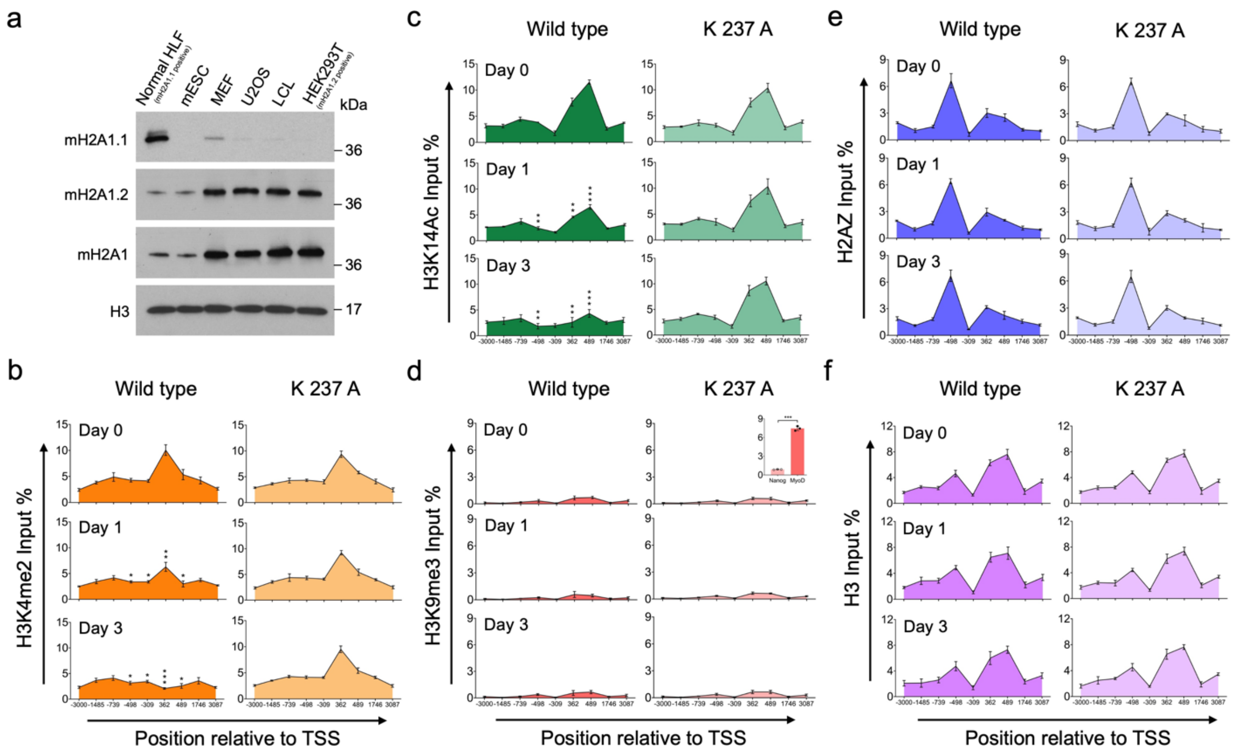

Supplementary Fig. 4: LSH recruitment induces dynamic changes of H3K4me2 and H3K14Ac enrichment at the tethered locus. a Western blot analysis for detection of macroH2A1.1, macroH2A1.2 and macroH2A1 in different cell types used in this study. Normal human lung fibroblast (HLF) and HEK293T cells were used as positive cells for macroH2A1.1 and macroH2A1.2, respectively. **b-f** ChIP-qPCR analysis to show H3K4me2 (**b**), H3K14Ac (**c**), H3K9me3 (**d**), H2A.Z (**e**) and H3 core histone (**f**) enrichment profiles at the *CiA: Oct4* locus in wild type LSH and ATP mutant LSH (K 237 A) CIP systems after 0, 1 and 3 days of rapamycin treatment (n = 3 independent experiments). *Nanog* and *MyoD* genes were used as negative and positive controls for H3K9me3 ChIP-qPCR results (n = 3 independent experiments). Data are represented as mean ± SD. Significance assessed using one-way ANOVA with Tukey's multiple comparison test (*adjusted $p < 0.05$, **adjusted $p < 0.01$ and ***adjusted $p < 0.001$). Source data are provided as a Source Data file.

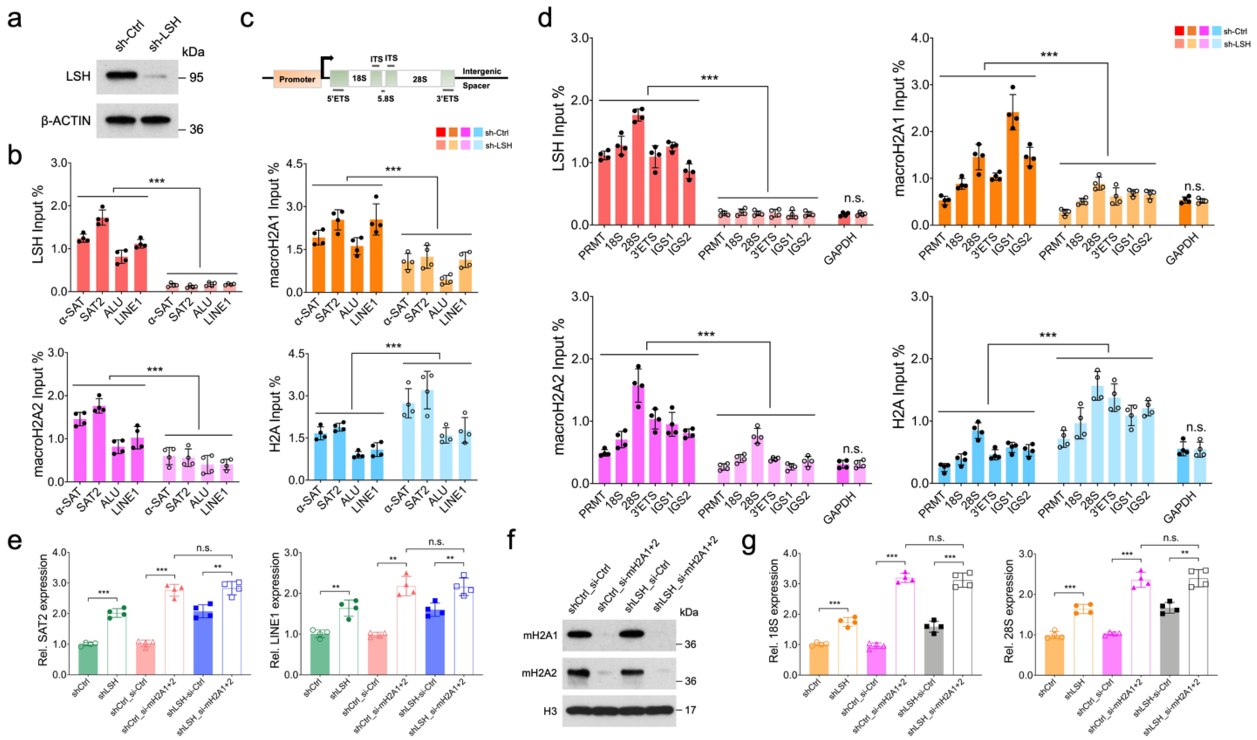

Supplementary Fig. 5: LSH knockdown leads to reduction of macroH2A enrichment at repeats and de-repression of transcripts in U2OS cells. a Western blot analysis for detection of LSH protein in sh-Ctrl and sh-*LSH* U2OS cells. **b** ChIP-qPCR analysis for detection of LSH, macroH2A1, macroH2A2 and H2A enrichment at repeat sequences including α-SAT, SAT2, ALU and LINE1 elements in sh-Ctrl and sh-*LSH* U2OS cells. ***$p < 0.0001$. **c-d** Schematic representation of human rDNA repeats (**c**). PRMT, promoter; ETS, external transcribed spacer; ITS, internal transcribed spacer; IGS, intergenic spacer. ChIP-qPCR analysis for detection of LSH, macroH2A1, macroH2A2 and H2A enrichment at rDNA in sh-Ctrl and sh-*LSH* U2OS cells (**d**). ***$p < 0.0001$. n.s. means not significant. **e-g** RT-qPCR analysis in sh-Ctrl and sh-*LSH* U2OS cells, or cells treated with control siRNA (si-Ctrl) or two combined macroH2A siRNA (si-mH2A1+2), in order to compare the effects of LSH and macroH2A depletion on repeats (SAT2 and LINE1, **e**) and rDNA (18S and 28S, **g**) transcriptional levels. Western blot analysis for determination of macroH2A depletion (**f**). **adjusted $p < 0.01$ and ***adjusted $p < 0.001$, n.s. means not significant. Data are represented as mean ± SD. Paired two-tailed Student's t test (**b**, **d**); one-way ANOVA with Tukey's multiple comparison test (**e**, **g**). (**b**, **d**, **e**, **g**) representative of four independent experiments. Source data are provided as a Source Data file.

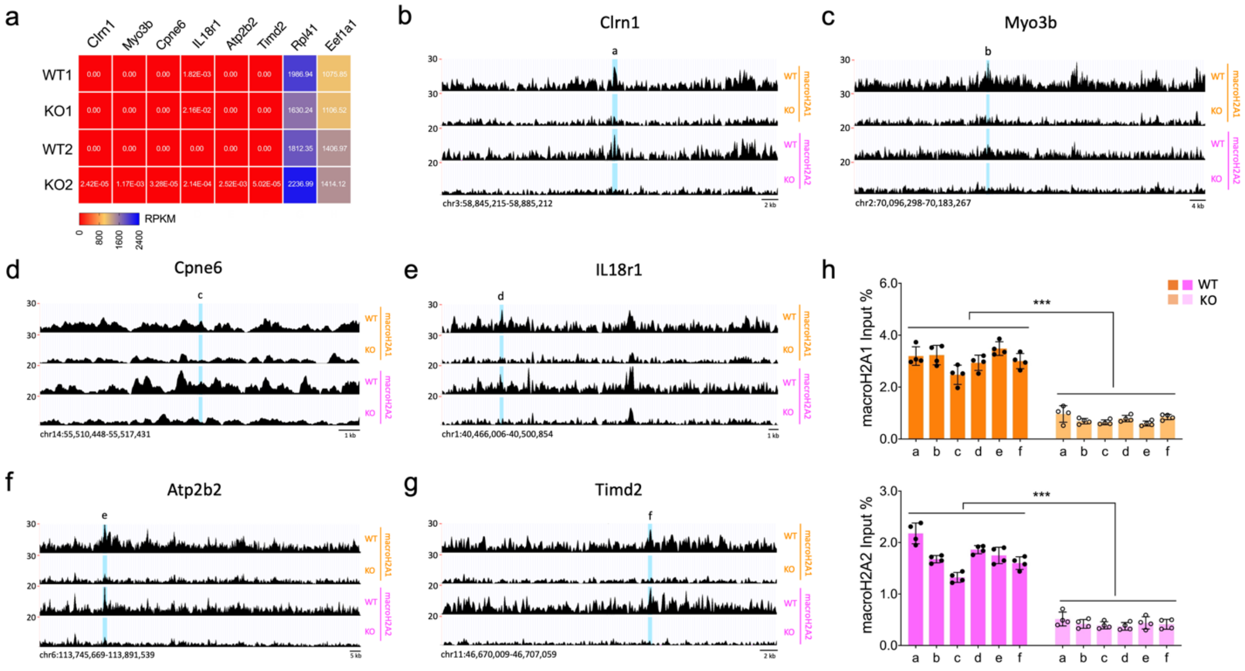

Supplementary Fig. 6: LSH alters macroH2A deposition at silenced genes. a Heatmap to illustrate the relative steady state RNA expression of the selected silenced genes in *Lsh* WT and KO MEFs. Two genes with high transcriptional level served as positive controls. **b-g** Genome browser snapshots to display macroH2A1 and macroH2A2 enrichment at the selected silenced genes in *Lsh* KO MEFs compared to WT MEFs. **h** The selected sites (a-f) at the silenced genes as shown (b-g) were used for validation of macroH2A occupancy level by ChIP-qPCR analysis (n = 4 independent experiments). Data are represented as mean ± SD. Significance assessed using paired two-tailed Student's t test (***$p < 0.0001$). Source data are provided as a Source Data file.

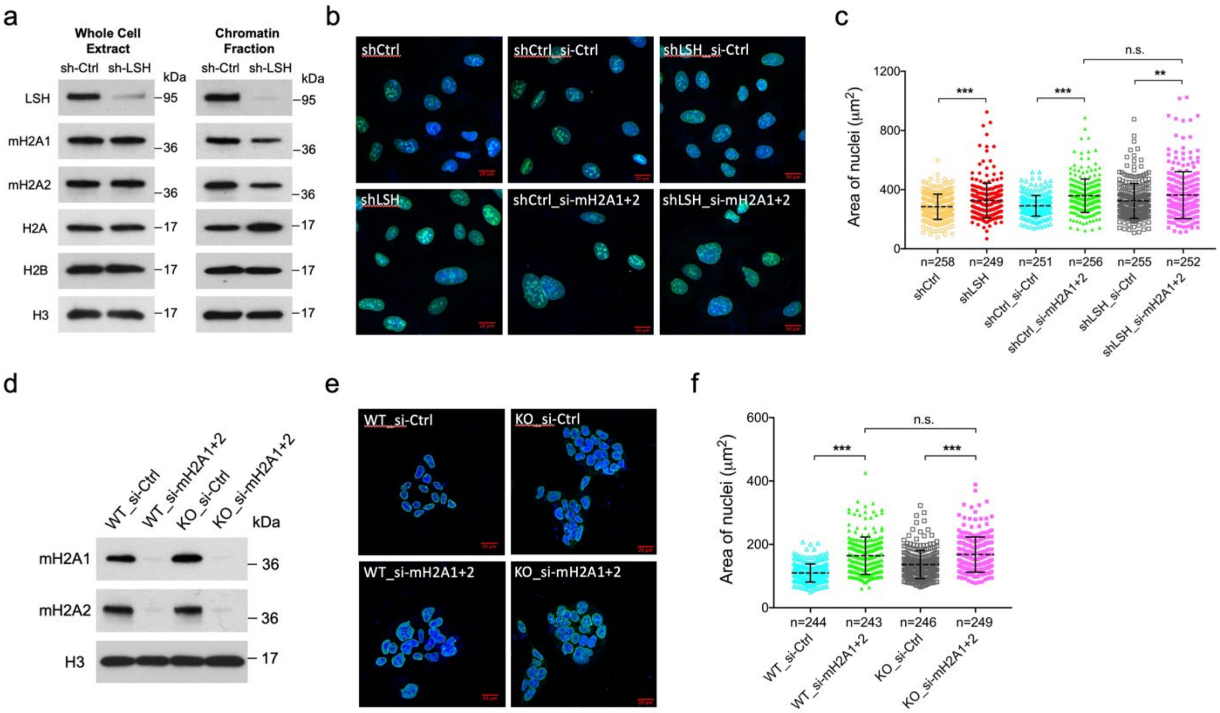

Supplementary Fig. 7: LSH knockdown leads to decreased chromatin association of macroH2A and to an increase of nuclear size. a Western blot analysis for detection of indicated proteins in whole cell extract or in chromatin fraction isolated from sh-Ctrl and sh-*LSH* U2OS cells. **b-c** IF assay of Lamin B1 and DAPI in sh-Ctrl and sh-*LSH* U2OS cells, or cells treated with control si-RNA (si-Ctrl) or two combined macroH2A siRNA (si-mH2A1+2), in order to measure nuclear area (**b**). Scale bar, 20 µm. The statistical analysis is shown in dot plot (**c**). **adjusted p < 0.01 and ***adjusted p < 0.001, n.s. means not significant. **d-f** Western blot analysis of WT and KO mouse ES cells treated with control si-RNA (si-Ctrl) or two combined macroH2A siRNA (si-mH2A1+2) for determination of macroH2A depletion (**d**). Afterwards, cells were stained with Lamin B1 and DAPI to visualize nuclear size (**e**). Scale bar, 20 µm. Quantification of nuclear area of samples is shown in **f**. ***adjusted p < 0.001, n.s. means not significant. Data are represented as dot plots with mean ± SD (**c**, **f**). One-way ANOVA with Tukey's multiple comparison test (**c**, **f**). Source data are provided as a Source Data file.

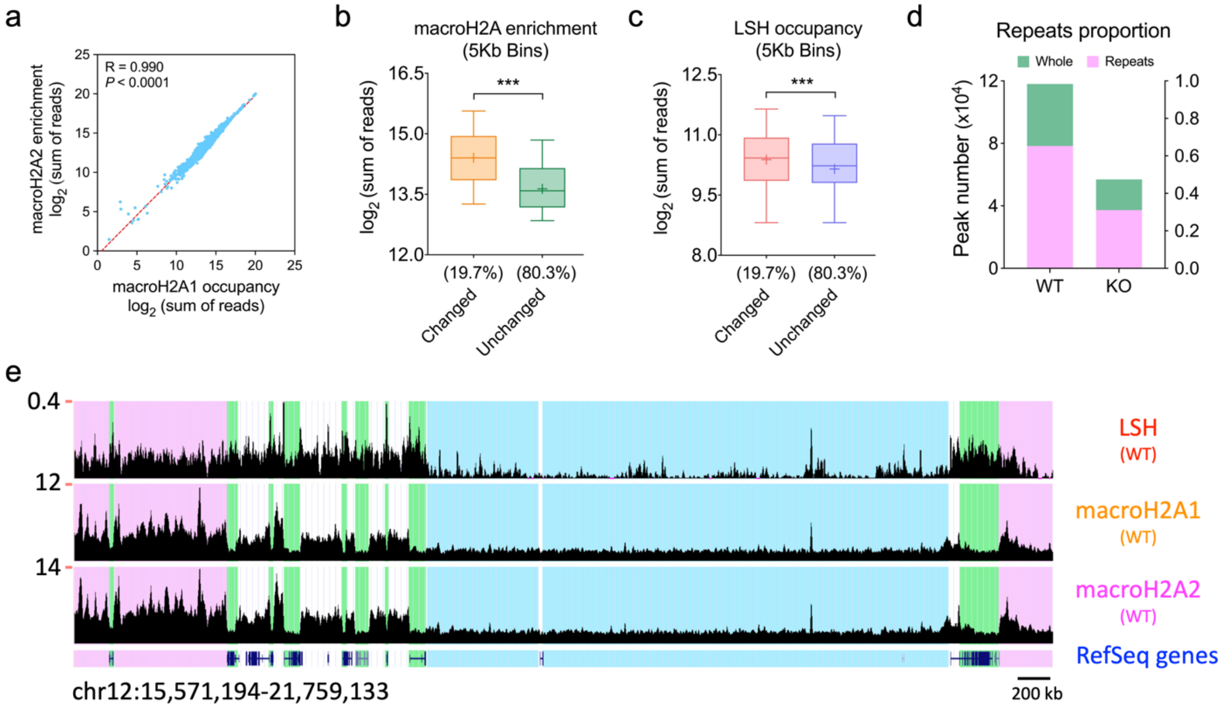

Supplementary Fig. 8: ChIP-seq analysis data supporting Fig. 5. a Scatterplot to demonstrate the correlation between macroH2A1 occupancy level (5Kb tiles) and macroH2A2 enrichment (5Kb tiles). The Pearson correlation coefficient is shown. **b-c** Boxplots representing macroH2A (**b**) and LSH (**c**) occupancy levels (divided in fixed 5Kb bins) at the sites in WT MEFs where macroH2A deposition is changed (19.7%, n = 94,727 bins) or unchanged (80.3%, n = 385,955 bins) by LSH depletion. Cross dots (+), sample mean; center lines, median; boxes, 25-75 percentiles; whiskers, 1.5 IQR. Outliers are not shown. Significance assessed using unpaired two-tailed Student's t test (***p < 0.0001). **d** Illustration of macroH2A peak overlapping proportion with repeat sequences in *Lsh* WT and KO MEF cells. **e** Genome browser snapshot to illustrate the role of LSH and transcriptional pruning. In gene-free regions low LSH occupancy is associated with low macroH2A deposition (blue) or high LSH binding is associated with high macroH2A occupancy (magenta); the presence or absence of LSH maybe the main determining factor in these regions, since these regions do not contain genes which could be transcribed. In gene dense regions, the broad domain with high LSH occupancy includes small regions with low macroH2A enrichment which correspond with the localization of genes (green). At these locations, transcriptional pruning may remove macroH2A. Source data are provided as a Source Data file.

Supplementary Table 1. Primers used in this study.

Primer	Direction	Sequence	Application
CiA:Oct4: -3000	F	ACCACAGCAGCCAAGAGATT	ChIP
	R	GTCCACCAGCATGAAAAGGT	ChIP
CiA:Oct4: -1485	F	CAGAGCATGGTGTAGGAGCA	ChIP
	R	GCTGGCGGAAAGACACTAAG	ChIP
CiA:Oct4: -739	F	GCACTTCTCTGGGGTCTCTG	ChIP
	R	ACCCACCCGTCTAGAGTCCT	ChIP
CiA:Oct4: -498/	F	TGATGGGCGGTGCAGTAA	ChIP
V5	R	CACGCCCATCATTACCACAA	ChIP
CiA:Oct4: -309/	F	GAGCTTGCATGCCTGCAGGT	ChIP
Binding site	R	CAGGAGGCCTTCATTTTCAA	ChIP
CiA:Oct4: 362/	F	CAGCGTGTCCGGCGAGGGCGA	ChIP
GFP site	R	TCCTTGAAGTCGATGCCCTTCAG	ChIP
CiA:Oct4: 489	F	CACATGAAGCAGCACGACTT	ChIP
	R	AGTTCACCTTGATGCCGTTC	ChIP
CiA:Oct4: 1746	F	CTGGCCCTTGTCATGTAGGT	ChIP
	R	GCCAGAGGCCACTTGTGTAG	ChIP
CiA:Oct4: 3087	F	CGTTGGCTACCCGTGATATT	ChIP
	R	CGCTTGGTCGGTCTTTATTC	ChIP
Ms Nanog	F	CAGACTGGGAGGGAGGGAAA	ChIP
	R	GAGGTGCAGCCGTGGTTAAA	ChIP
Ms MyoD	F	CTCCCACCTCCACTGACATT	ChIP
	R	ATGTCCAGGGGGTAACTGTG	ChIP
Ms Major	F	AAATACACACTTTAGGACG	ChIP
	R	TCAAGTGGATGTTTCTCATT	ChIP
Ms LINE1	F	TTCTTCTTCGGCCAGGAGGAGGTC	ChIP
	R	GCTGGGTTCTGGTGATGGTGAGTG	ChIP
Ms IAP	F	CAAATAATCTGCGCATATGCCGA	ChIP
	R	GACCAGAATCTTCTGCGGCAA	ChIP
Ms MINOR	F	TTGTACAACAGTGTATATCAATG	ChIP
	R	GTTTCCAACGAATGTGTTTTTCAG	ChIP
Ms ENHC	F	TACTTCTGAGGCCGAGAGGA	ChIP
	R	GATCCAAAGCTCCAGCTGAC	ChIP
Ms PRMT	F	CCTTTGAGGTCCGGTTCTTT	ChIP
	R	TCCAGGTCCAATAGGAACAGAT	ChIP
Ms 5’ETS	F	ACTGACACGCTGTCCTTTCC	ChIP
	R	CGACAGACCCAAGCCAGTA	ChIP
Ms 18S	F	GTGGAGCGATTTGTCTGGTT	ChIP/RT-qPCR
	R	CGCTGAGCCAGTCAGTGTAG	ChIP/RT-qPCR
Ms 5.8s	F	GACTCTTAGCGGTGGATCACTC	ChIP
	R	GACGCTCAGACAGGCGTAG	ChIP

Ms ITS	F	GTGTCGTTCCCGTGTTTTTC	ChIP
	R	ATCGGTATTTCGGGTGTGAG	ChIP
Ms 28S	F	AAATGTGGCGTACGGAAGAC	ChIP/RT-qPCR
	R	CGTGCCGGTATTTAGCCTTA	ChIP/RT-qPCR
Ms IGS1	F	TCTTCCGAAGGTGCAGAGTT	ChIP
	R	TCCTCCTCCTCCTCCTCTTC	ChIP
Ms IGS2	F	CTTCCCAAATGCTGGGATTA	ChIP
	R	AAGGCAGCTAGGGCTACACA	ChIP
Ms Gapdh	F	GCACCACCAACTGCTTAG	ChIP
	R	GATGCAGGGATGATGTTC	ChIP
High_I	F	CCCAACTCTGCAGTCTCCAT	ChIP
	R	GAAAAGCTCAGGGCAAAGTG	ChIP
High_II	F	TTGGTCTGCCAAAGGTAAGG	ChIP
	R	GAGAGGCCTCCAGATGTCAG	ChIP
High_III	F	CAGCAGCCCTCAAAGATTTC	ChIP
	R	ACAGGTAATGCATGCCAACA	ChIP
High_IV	F	AATAGGCAGGCCAATGACAC	ChIP
	R	ACGAAAGAAGAGGCACTGGA	ChIP
High_V	F	GTTTTACTGCCGTCCTGCTC	ChIP
	R	GTGAGGCCGTTTGGAAATTA	ChIP
Low_VI	F	TGATAAGCCACCATGACCAA	ChIP
	R	GGGGATGGGTTTTCAAGTTT	ChIP
Low_VII	F	GTGCCTTGACCTGACACTCA	ChIP
	R	GTGGGCCAAGTCTCCATTTA	ChIP
Low_VIII	F	CATTGGACACGCAGACTTTG	ChIP
	R	TGAATGTCCTGAGGAAAACTGA	ChIP
Low_IX	F	TGAGATTCCACCTCACACCA	ChIP
	R	TTACCAGTTGGGGCATCTTC	ChIP
Low_X	F	CGGAAACCACACCCTAGAAA	ChIP
	R	GCAGAATGTTGGGTCCTGTT	ChIP
Hu PRMT	F	CTCCCGCTCTGGAGACAC	ChIP
	R	GGACACCTGTCCCCAAAAAC	ChIP
Hu 18s	F	GGATGCGTGCATTTATCAGA	ChIP
	R	GATCGGCCCGAGGTTATCTA	ChIP
Hu 28s	F	AGTCGGTTGCTTGGGAATGC	ChIP
	R	CCCTTACGGTACTTGTTGACT	ChIP
Hu 3'ETS	F	ACCTGGCGCTAAACCATTCGT	ChIP
	R	GGACAAACCCTTGTGTCGAGG	ChIP
Hu IGS1	F	GTTGACGTACAGGGTGGACTG	ChIP
	R	GGAAGTTGTCTTCACGCCTG	ChIP
Hu IGS2	F	CCTTCCACGAGAGTGAGAAGCG	ChIP
	R	CTCGACCTCCCGAAATCGTACA	ChIP
Hu GAPDH	F	TCCACCACCCTGTTGCTGTA	ChIP

	R	ACCACAGTCCATGCCATCAC	ChIP
Hu α-SAT	F	TCATTCCCACAAACTGCGTTG	ChIP
	R	TCCAACGAAGGCCACAAGA	ChIP
Hu SAT2	F	CATCGAATGGAAATGAAAGGAGTC	ChIP/RT-qPCR
	R	ACCATTGGATGATTGCAGTCAA	ChIP/RT-qPCR
Hu ALU	F	CCAGAAAAAATTACCAATTAGTTC	ChIP
	R	GGGCCTATTGACTATGCTTAC	ChIP
Hu LINE1	F	AGCCTAACTGGGAGGCACCC	ChIP
	R	GATGATGGTGATGTACAGATGGG	ChIP
Ms Clrn1_a	F	AGTGCAGGTCCACGAGTTTT	ChIP
	R	ACACTGTGGACAGGGAGACC	ChIP
Ms Myo3b_b	F	TCTTGGGCAGGAAGAAGAAA	ChIP
	R	AATAGGCATGCATGGTCACA	ChIP
Ms Cpne6_c	F	AAGGTAAAGCCCTGGAGCAT	ChIP
	R	CCAGGAGCCACCTGATAAAC	ChIP
Ms IL18r1_d	F	ACTGCTCCTGCTTGCTGTTT	ChIP
	R	GCAGGCGAATTTCTGAGTTC	ChIP
Ms Atp2b2_e	F	AGCCACAGTCTGCCATCTCT	ChIP
	R	GGTTCTCAAGAGGCTTGTGC	ChIP
Ms Timd2_f	F	AGAGGCTGAAAGGAGGAAGG	ChIP
	R	CGACCCTGAGGATTTGGTTA	ChIP
Ms LINE1	F	AGATCTGGAACCATAGATG	RT-qPCR
	R	TTCTCATTGTGTCCTGGATT	RT-qPCR
Ms IAP	F	GCTCCGGTAGAA	RT-qPCR
	R	TGCCATGCCGGCGAGCCTGT	RT-qPCR
GFP	F	CTGGTCGAGCTGGACGGCGACG	RT-qPCR
	R	CACGAACTCCAGCAGGACCATG	RT-qPCR
Ms Oct4	F	TGGCGTGGAGACTTTGCA	RT-qPCR
	R	GAGGTTCCCTCTGAGTTGCTTTC	RT-qPCR
Ms Nanog	F	TTCCTGGTCCCCACAGTTTG	RT-qPCR
	R	CACTGGTTTTTCTGCCACCG	RT-qPCR
Ms Sox2	F	GGAGAACCCCAAGATGCACA	RT-qPCR
	R	CGAGCTGGTCATGGAGTTGT	RT-qPCR
Ms Gapdh	F	TCCATGACAACTTTGGCATTG	RT-qPCR
	R	CAGTCTTCTGGGTGGCAGTGA	RT-qPCR
Hu 18S	F	CTCTAGATAACCTCGGGCCG	RT-qPCR
	R	GTCGGGAGTGGGTAA TTTGC	RT-qPCR
Hu 28S	F	GGTGTTGACGCGATGTGATT	RT-qPCR
	R	GCTGTGGTTTCGCTGGATAG	RT-qPCR
Hu LINE1	F	TAACCAATACAGAGAAGTGC	RT-qPCR
	R	GATAATATCCTGCAGAGTGT	RT-qPCR
Hu GAPDH	F	GAGTCAACGGATTTGGTCGT	RT-qPCR
	R	TTGATTTTGGAGGGATCTCG	RT-qPCR

GFP	F	TGGGGTATAAGTTGGAGTATAATTATAATA	Bisulfite PCR
	R	AACTCCAACAAAACCATATAATC	Bisulfite PCR
Ms IAP	F	TTGTGTTTTAAGTGGTAAATAAATAATTTG	Bisulfite PCR
	R	CAAAAAAAACACACAAACCAAAAT	Bisulfite PCR

Supplementary Table 2. Summary of nChIP-seq data generated in this study.

Sample_ID	Cell description	nChIP	Total reads	Mapped reads	Percentage map
KO1_macroH2A1	KO1 MEF	macroH2A1	106046394	101704497	95.91%
KO1RS_macroH2A1	KO2 MEF	macroH2A1	60311698	58734602	97.39%
KO2RS_macroH2A1	KO3 MEF	macroH2A1	64242828	62662046	97.54%
KO6_macroH2A2	KO1 MEF	macroH2A2	73396866	72082858	98.21%
KO7_macroH2A2	KO2 MEF	macroH2A2	81260704	79708797	98.09%
KO8_macroH2A2	KO3 MEF	macroH2A2	65589307	64417740	98.21%
KO1_macroH2A1	KO1 MEF	Input	107588112	105289915	97.86%
KO1RS_macroH2A1	KO2 MEF	Input	62172490	60879651	97.92%
KO2RS_macroH2A1	KO3 MEF	Input	51900612	50816466	97.91%
KO6_macroH2A2	KO1 MEF	Input	82684289	81335997	98.37%
KO7_macroH2A2	KO2 MEF	Input	73154971	71921345	98.31%
KO8_macroH2A2	KO3 MEF	Input	34537094	33994260	98.43%
KO1_H2B	KO1 MEF	H2B	40194541	38794270	96.52%
KO2_H2B	KO2 MEF	H2B	46125111	44541659	96.57%
KO3_H2B	KO3 MEF	H2B	47735443	46046527	96.46%
KO1_H2B	KO1 MEF	Input	40763729	39875985	97.82%
KO2_H2B	KO2 MEF	Input	63040158	61773980	97.99%
KO3_H2B	KO3 MEF	Input	39676607	38839072	97.89%
WT1_macroH2A1	WT1 MEF	macroH2A1	139656033	134899826	96.59%
WT1RS_macroH2A1	WT2 MEF	macroH2A1	58027460	56900901	98.06%
WT2RS_macroH2A1	WT3 MEF	macroH2A1	61056336	59733591	97.83%
WT6_macroH2A2	WT1 MEF	macroH2A2	72249028	70314571	97.32%
WT7_macroH2A2	WT2 MEF	macroH2A2	69677821	68454534	98.24%
WT8_macroH2A2	WT3 MEF	macroH2A2	80782526	79198476	98.04%
WT1_macroH2A1	WT1 MEF	Input	137490159	134773802	98.02%
WT1RS_macroH2A1	WT2 MEF	Input	59726894	58669919	98.23%
WT2RS_macroH2A1	WT3 MEF	Input	60112986	59084083	98.29%
WT6_macroH2A2	WT1 MEF	Input	72121925	70901519	98.31%
WT7_macroH2A2	WT2 MEF	Input	66689529	65649822	98.44%
WT8_macroH2A2	WT3 MEF	Input	81734396	80459689	98.44%
WT1_H2B	WT1 MEF	H2B	47257584	45665005	96.63%
WT2_H2B	WT2 MEF	H2B	42959036	41476511	96.55%
WT3_H2B	WT3 MEF	H2B	39911743	38531071	96.54%
WT1_H2B	WT1 MEF	Input	42266124	41403609	97.96%
WT2_H2B	WT2 MEF	Input	47452859	46531099	98.06%
WT3_H2B	WT3 MEF	Input	53206474	52134315	97.98%

Supplementary Table 3. Antibodies used in this study.

Antibody	Company	Catalog #	Dilution for WB	Dilution for IF	CO-IP(μg)	ChIP (μg)
V5	Thermo Fisher	R960-25	1:1000	1:200		3 μg
HA	Thermo Fisher	26183	1:1000	1:200		
Flag	Thermo Fisher	MA1-91878	1:1000		4 μg	
GFP	Thermo Fisher	A-6455	1:1000			
GFP	Thermo Fisher	A-11120			4 μg	
LSH	Kathrin lab		1:5000			3 μg
RNA polymerase II	Millipore	05-623				3 μg
H3K4me2	abcam	ab7766				3 μg
H3K4me3	abcam	ab8580				3 μg
H3K14Ac	abcam	ab52946				3 μg
H3K27Ac	abcam	ab4729				3 μg
H3K9me3	abcam	ab8898				3 μg
H3K27me3	abcam	ab6002				3 μg
H1.2	abcam	ab4086				3 μg
macroH2A1	abcam	ab37264				3 μg
macroH2A1	abcam	ab183041	1:1000			
macroH2A2	abcam	ab102126	1:1000			3 μg
macroH2A1.1	Cell signaling	12455S	1:500			
macroH2A1.2	Cell signaling	4827S	1:500			
H2A	abcam	ab18255	1:2000			3 μg
H3	abcam	ab1791	1:5000			3 μg
H2B	abcam	ab1790	1:2000			3 μg
H2AZ	abcam	ab4174	1:1000			3 μg
OCT4	Cell signaling	2750S	1:1000			
β-ACTIN	Thermo Fisher	MA1-140	1:5000			
Lamin B1	abcam	ab16048		1:200		
Rabbit IgG	Millipore	12-370				3 μg
Mouse IgG	Millipore	12-371				3 μg
anti-rabbit IgG, HRP	abcam	ab6721	1:2500			
anti-mouse IgG, HRP	abcam	ab6728	1:2500			
anti-rabbit IgG, 488	abcam	ab150077		1:500		
anti-rabbit IgG, 594	abcam	ab150080		1:500		
anti-mouse IgG, 488	abcam	ab150113		1:500		
anti-mouse IgG, 594	abcam	ab150116		1:500		
