# Supplementary material for: LSH mediates gene repression through macroH2A deposition
Source: Nat Commun. 2020 Nov 6;11:5647. doi: 10.1038/s41467-020-19159-0 (PMC7648012; doi:10.1038/s41467-020-19159-0)
Supplement: Supplementary file 3 — Reporting Summary [file 41467_2020_19159_MOESM3_ESM.pdf]

nature research

Corresponding author(s): Kathrin Muegge

Last updated by author(s): Aug 18, 2020

Reporting Summary

Nature Research wishes to improve the reproducibility of the work that we publish. This form provides structure for consistency and transparency in reporting. For further information on Nature Research policies, see Authors & Referees and the Editorial Policy Checklist .

Statistics

For all statistical analyses, confirm that the following items are present in the figure legend, table legend, main text, or Methods section.

n/a	Confirmed	
☐	☒	The exact sample size (*n*) for each experimental group/condition, given as a discrete number and unit of measurement
☐	☒	A statement on whether measurements were taken from distinct samples or whether the same sample was measured repeatedly
☐	☒	The statistical test(s) used AND whether they are one- or two-sided<br>*Only common tests should be described solely by name; describe more complex techniques in the Methods section.*
☒	☐	A description of all covariates tested
☒	☐	A description of any assumptions or corrections, such as tests of normality and adjustment for multiple comparisons
☐	☒	A full description of the statistical parameters including central tendency (e.g. means) or other basic estimates (e.g. regression coefficient) AND variation (e.g. standard deviation) or associated estimates of uncertainty (e.g. confidence intervals)
☐	☒	For null hypothesis testing, the test statistic (e.g. *F*, *t*, *r*) with confidence intervals, effect sizes, degrees of freedom and *P* value noted<br>*Give P values as exact values whenever suitable.*
☒	☐	For Bayesian analysis, information on the choice of priors and Markov chain Monte Carlo settings
☒	☐	For hierarchical and complex designs, identification of the appropriate level for tests and full reporting of outcomes
☐	☒	Estimates of effect sizes (e.g. Cohen's *d*, Pearson's *r*), indicating how they were calculated

Our web collection on statistics for biologists contains articles on many of the points above.

Software and code

Policy information about availability of computer code

Data collection	For data collection no software was used.
Data analysis	epic2 v. 0.0.41; samtools v. 1.10; MACS v. 2.2.6; wigToBigWig v. 4; Bedops v. 2.4.39; Blat program from UCSC toolkit v. 367; BiQ analyzer v. 0.7; RepeatMasker version v. 4.1.0; Excel Microsoft 365 ProPlus; Graphpad prism v. 8; FlowJo software v. 10; ImageJ software v. 2; RTA software 1.8.70.0; Cutadapt 1.18; Bowtie2 2.2.6.

For manuscripts utilizing custom algorithms or software that are central to the research but not yet described in published literature, software must be made available to editors/reviewers. We strongly encourage code deposition in a community repository (e.g. GitHub). See the Nature Research guidelines for submitting code & software for further information.

Data

Policy information about availability of data

All manuscripts must include a data availability statement. This statement should provide the following information, where applicable:
- Accession codes, unique identifiers, or web links for publicly available datasets
- A list of figures that have associated raw data
- A description of any restrictions on data availability

Source data are provided with this paper and its supplementary information files and are available from the corresponding author upon reasonable request. A reporting summary for this article is available as a Supplementary Information file. The source data underlying all figures are provided as a Source Data file. RNA-seq data was retrieved from GEO database (GSM1382355, GSM1382356, GSM1382357 and GSM1382358). LSH ChIP-seq data in WT MEFs was retrieved from GEO database (GSM835828). The macroH2A and H2B ChIP-seq data has been deposited in Gene Expression Omnibus, GEO accession number: GSE142082.

Field-specific reporting

Please select the one below that is the best fit for your research. If you are not sure, read the appropriate sections before making your selection.

- [x] Life sciences
- [ ] Behavioural & social sciences
- [ ] Ecological, evolutionary & environmental sciences

For a reference copy of the document with all sections, see nature.com/documents/nr-reporting-summary-flat.pdf

Life sciences study design

All studies must disclose on these points even when the disclosure is negative.

Sample size	Sample size was not predetermined by statistical method as this work did not involve animal models or human subjects. All high-throughput sequencing assays were performed in triplicates using independent biological (cell culture) samples derived from individual embryos. All mutants were selected in advance. For all other assays, sample size was decided in advance, and uniform.
Data exclusions	No data was excluded.
Replication	Each experiment, including ChIP-seq samples, includes at least three independent replicates to conduct statistical analysis. ChIP-seq samples as well as their respective Input samples were derived as biologic replicates from three murine embryonic fibroblast cell lines for each genotype. Each cell line was derived from an individual embryo. All immunoblots were repeated at least three times with different biological samples and led to similar results. All attempts at replication for all other assays were successful.
Randomization	No human participants or animal models were reported in this manuscript. Experimental groups were selected based on the individual genetic variant (WT, KO or Lsh mutants). Appropriate controls were included for each experiment, such as no rapamycin treatment and no modified Oct4 allele (tethering assay), normal IgG control (ChIPs), cells transfected with control shRNA or control siRNA, Input DNA and H2B (ChIp-seq).
Blinding	Data sets were not analyzed blindly, but were all processed according to uniform and identical processing steps.

Reporting for specific materials, systems and methods

We require information from authors about some types of materials, experimental systems and methods used in many studies. Here, indicate whether each material, system or method listed is relevant to your study. If you are not sure if a list item applies to your research, read the appropriate section before selecting a response.

Materials & experimental systems

n/a	Involved in the study	
☐	☒	Antibodies
☐	☒	Eukaryotic cell lines
☒	☐	Palaeontology
☒	☐	Animals and other organisms
☒	☐	Human research participants
☒	☐	Clinical data

Methods

n/a	Involved in the study	
☐	☒	ChIP-seq
☐	☒	Flow cytometry
☒	☐	MRI-based neuroimaging

Antibodies

Antibodies used

V5, Thermo Fisher, Cat.# R960-25;
HA, Thermo Fisher, clone 2-2.2.14, Cat.# 26183;
Flag, Thermo Fisher, clone FG4R, Cat.# MA1-91878;
GFP, Thermo Fisher, Cat.# A-6455;
GFP, Thermo Fisher, clone 3E6, Cat.# A-11120;
LSH, generated by Dr. Kathrin Muegge's lab, polyclonal rabbit-antiserum raised against recombinant LSH;
RNA polymerase II, Millipore, clone CTD4H8, Cat.# 05-623;
H3K4me2, abcam, Cat.# ab7766;
H3K4me3, abcam, Cat.# ab8580;
H3K14Ac , abcam, clone EP964Y, Cat.# ab52946;
H3K27Ac, abcam, Cat.# ab4729;
H3K9me3, abcam, Cat.# ab8898;
H3K27me3, abcam, Cat.# ab6002;
H1.2, abcam, Cat.# ab4086;
H2AZ, abcam, Cat. # ab4174;
macroH2A1, abcam, Cat.# ab37264;
macroH2A1, abcam, clone EPR9359(2), Cat.# ab183041;

macroH2A2, abcam, Cat.# ab102126;
macroH2A1.1, Cell signaling, clone D5F6N, Cat.# 12455S;
macroH2A1.2, Cell signaling, Cat.# 4827S;
H2A, abcam, Cat.# ab18255;
H3, abcam, Cat.# ab1791;
H2B, abcam, Cat.# ab1790;
OCT4, Cell Signaling, Cat.# 2750S;
β-ACTIN, Thermo Fisher, clone 15G5A11/E2, Cat.# MA1-140;
Lamin B1, abcam, Cat.# ab16048;
Rabbit IgG, Millipore, Cat.# 12-370;
Mouse IgG, Millipore, Cat.# 12-371;
anti-rabbit IgG, HRP, abcam, Cat.# ab6721
anti-mouse IgG, HRP, abcam, Cat.# ab6728
anti-rabbit IgG, 488 abcam, Cat.# ab150077
anti-rabbit IgG, 594 abcam, Cat.# ab150080
anti-mouse IgG, 488 abcam, Cat.# ab150113
anti-mouse IgG, 594 abcam, Cat.# ab150116

Validation

Primary antibodies were validated as follows:
1. Anti-V5 (Mouse, Thermo Fisher, R960-25): validated by the manufacturer for application of WB, ELISA, ICC, IF and IP to detect V5 Tag with 901 references. Published species reactivity: Bacteria, Bovine, C. elegans, Fruit fly, Hamster, Human, Insect, Marsupial, Mouse, Non-human primate, Pig, Protozoa, Rat, Tag, Virus, Yeast.
2. Anti-HA (Mouse, Thermo Fisher, 26183): validated by the manufacturer for application of ICC, IF, IP and WB to detect HA tag with 62 references. Published species reactivity: Human.
3. Anti-Flag (Mouse, Thermo Fisher, MA1-91878): validated by the manufacturer for application of ICC, IF, IP and WB to detect Flag tag with 58 references. Published species reactivity: Amphibian, Human.
4. Anti-GFP (Rabbit, Thermo Fisher, A-6455): validated by the manufacturer for application of ELISA, ICC, IF and IHC to detect GFP tag with 1,005 references. Published species reactivity: Chimpanzee, Dog, Fish, Fruit fly, Hamster, Human, Marsupial, Mouse, Non-human primate, Rat, Tag, Xenopus.
5. Anti-GFP (Mouse, Thermo Fisher, A-11120): validated by the manufacturer for application of ELISA, ICC, IF and IP to detect GFP tag with 583 references. Published species reactivity: C. elegans, Fruit fly, Human, Insect, Mouse, Non-human primate, Rat, Tag, Zebrafish.
6. Anti-LSH (Rabbit, Dr. Kathrin Muegge's lab): validated in Dr. Kathrin Muegge's lab for application of WB, IF, IP, IHC, ICC and ChIP to detect LSH with the references: He Y et al, PNAS, 2020; Ren J et al, Epigenetics, 2019; Ren J et al, Epigenetics, 2018; Ren J et al, Nucleic Acids Res, 2015, etc. Published species reactivity: mouse, human.
7. Anti-RNA polymerase II (Mouse, Millipore, 05-623): validated by the manufacturer for application of WB, ChIP-seq to detect Phospho- and non-phospho-RNA polymerase II with 223 references. Published species reactivity: Human, Rat, Mouse, Yeast.
8. Anti-H3K4me2 (Rabbit, abcam, ab7766): validated by the manufacturer for application of ChIP, WB, PepArr, ICC/IF to detect Histone H3 (di methyl K4) with 243 references. Published species reactivity: Mouse, Human, Pig, Saccharomyces cerevisiae, Tetrahymena, Drosophila melanogaster, Schizosaccharomyces pombe, Plasmodium falciparum, Common marmoset.
9. Anti-H3K4me3 (Rabbit, abcam, ab8580): validated by the manufacturer for application of PepArr, ChIP, WB, ICC/IF to detect Histone H3 (tri methyl K4) with 1455 references. Published species reactivity: Mouse, Rat, Rabbit, Human, Pig, Saccharomyces cerevisiae, Tetrahymena, Arabidopsis thaliana, Caenorhabditis elegans, Drosophila melanogaster, Trypanosoma cruzi, Common marmoset, Rice, Xenopus tropicalis.
10. Anti-H3K14Ac (Rabbit, abcam, ab52946): validated by the manufacturer for application of ChIP, WB, IHC-P, ICC/IF to detect Histone H3 (acetyl K14) with 62 references. Published species reactivity: Rat, Human.
11. Anti-H3K27Ac (Rabbit, abcam, ab4729): validated by the manufacturer for application of ICC/IF, WB, IHC-P, ChIP, PepArr to detect Histone H3 (acetyl K27) with 1068 references. Published species reactivity: Mouse, Rat, Cow, Arabidopsis thaliana, Drosophila melanogaster, Plasmodium falciparum.
12. Anti-H3K9me3 (Rabbit, abcam, ab8898): validated by the manufacturer for application of IHC-P, ICC/IF, ChIP to detect Histone H3 (tri methyl K9) with 1121 references. Published species reactivity: Mouse, Rat, Human, Saccharomyces cerevisiae, Indian muntjac.
13. Anti-H3K27me3 (Mouse, abcam, ab6002): validated by the manufacturer for application of ChIP, ELISA, WB, IHC - Wholemount, ICC/IF to detect Histone H3 (tri methyl K27) with 628 references. Published species reactivity: Mouse, Cow, Human, Drosophila melanogaster, Plants, Zebrafish.
14. Anti-H1.2 (Rabbit, abcam, ab4086): validated by the manufacturer for application of ChIP, WB, IP, ICC/IF, IHC-P to detect Histone H1.2 with 15 references. Published species reactivity: Human.
15. Anti-macroH2A1 (Rabbit, abcam, ab37264): validated by the manufacturer for application of WB, IHC-P, ICC/IF to detect macroH2A1 with 19 references. Published species reactivity: Mouse, Human.
16. Anti-macroH2A1 (Rabbit, abcam, ab183041): validated by the manufacturer for application of WB, IHC-P, ICC/IF to detect macroH2A1 with 5 references. Published species reactivity: Mouse, Rat, Human.
17. Anti-macroH2A2 (Rabbit, abcam, ab102126): validated by the manufacturer and reviewers for application of WB, IHC-P, ChIP to detect macroH2A2 with 1 references. Published species reactivity: Mouse, Human.
18. Anti-macroH2A1.1 (Rabbit, Cell signaling, 12455S): validated by the manufacturer for application of WB, IF to detect macroH2A1.1 with 7 references. Published species reactivity: Mouse, Rat, Human.
19. Anti-macroH2A1.2 (Rabbit, Cell signaling, 4827S): validated by the manufacturer for application of WB, IF to detect macroH2A1.2 with 11 references. Published species reactivity: Mouse, Rat, Human, Monkey.
20. Anti-H2A (Rabbit, abcam, ab18255): validated by the manufacturer for application of ICC/IF, IHC-P, WB, IP, ChIP to detect H2A with 88 references. Published species reactivity: Mouse, Human.

21. Anti-H3 (Rabbit, abcam, ab1791): validated by the manufacturer for application of IHC-P, Electron Microscopy, ChIP, IP, WB to detect H3 with 3065 references. Published species reactivity: Mouse, Rat, Chicken, Dog, Human, Saccharomyces cerevisiae, Xenopus laevis, Arabidopsis thaliana, Caenorhabditis elegans, Drosophila melanogaster, Indian muntjac, Schizosaccharomyces pombe, Trypanosoma cruzi, Neurospora crassa, Toxoplasma gondii, Schistosoma mansoni.
22. Anti-H2B (Rabbit, abcam, ab1790): validated by the manufacturer for application of WB, IHC-P, ChIP, ICC/IF, IP to detect H2B with 160 references. Published species reactivity: Mouse, Rat, Chicken, Cow, Human, Saccharomyces cerevisiae, Xenopus laevis, Arabidopsis thaliana.
23. Anti-H2AZ (Rabbit, abcam, ab4174): validated by the manufacturer for application of ICC/IF, ChIP, WB to detect H2AZ with 118 references. Published species reactivity: Mouse, Rat, Cow, Human.
24. Anti-OCT4 (Rabbit, Cell signaling, 2750S): validated by the manufacturer for application of WB, IHC, IF, F, ChIP to detect OCT4 with 127 references. Published species reactivity: Mouse, Human.
25. Anti-β-ACTIN (Mouse, Thermo Fisher, MA1-140): validated by the manufacturer for application of ICC, IF, IP and WB to detect β-ACTIN with 14 references. Published species reactivity: Human, Mouse, Rat.
26. Anti-Lamin B1 (Rabbit, abcam, ab16048): validated by the manufacturer for application of ICC/IF, WB, IHC-P to detect Lamin B1 with 625 references. Published species reactivity: Mouse, Rat, Human, Indian muntjac.

Eukaryotic cell lines

Policy information about cell lines

Cell line source(s)

1. The CiA:Oct4 mouse ES cell line was provided from Dr. Gerald Crabtree's lab.
2. The WT (Lsh+/+), KO (Lsh-/-), KO+LSH and KO+LSH(K237A) mouse ES cell lines were generated in Dr. Kathrin Muegge's lab.
3. Primary WT (Lsh+/+) and KO (Lsh-/-) MEF cells were derived from day 13.5 embryos of Hells+/- heterozygotic matings and represent littermates, which were immortalized by SV40 large T antigen afterwards in Dr. Kathrin Muegge's lab.
4. U2OS and HEK293T cell lines were purchased from ATCC company.
5. Normal human lung fibroblast cell line was purchased from Coriell Institute for Medical Research.
6. The ICF4 parent and patient lymphoblastoid cell lines were provided from Dr. Claire Francastel's lab.

Authentication

1. The CiA:Oct4 mouse ES cell line was authenticated in Hathaway NA et al. Dynamics and Memory of Heterochromatin in Living Cells. Cell. 2012 Jun 22;149(7):1447-60.
2. The WT (Lsh+/+, KO (Lsh-/-), KO+LSH and KO+LSH(K237A) mouse ES cell lines were authenticated in Ren J et al. The ATP binding site of the chromatin remodeling homolog Lsh is required for nucleosome density and de novo DNA methylation at repeat sequences. Nucleic Acids Res. 2015 Feb 18;43(3):1444-55.
3. The WT (Lsh+/+) and KO (Lsh-/-) MEF cell lines were authenticated by genotyping PCR and WB.
4. U2OS cell line was authenticated by ATCC (ATCC® HTB-96™) with cell morphology, antigen expression, gene expression, DNA profile and cytogenetic analysis.
5. HEK293T cell line was authenticated by ATCC (ATCC® CRL-11268™) with cell morphology, antigen expression and gene expression analysis.
6. Normal human lung fibroblast cell line was authenticated by Coriell Institute for Medical Research (AG06814-J) with confirmation of clinical summary, cell morphology, nucleoside phosphorylase, glucose-6-phosphate dehydrogenase, lactate dehydrogenase Isoenzyme electrophoresis and chromosome analysis.
7. The ICF4 parent and patient lymphoblastoid cell lines were authenticated in Thijssen PE et al. Mutations in CDCA7 and HELLS cause immunodeficiency-centromeric instability-facial anomalies syndrome. Nat Commun. 2015 Jul 28;6:7870.

Mycoplasma contamination

Cell lines were tested negative for mycoplasma contamination.

Commonly misidentified lines
(See ICLAC register)

No commonly misidentified cell lines were used in this study.

ChIP-seq

Data deposition

- [x] Confirm that both raw and final processed data have been deposited in a public database such as GEO.
- [x] Confirm that you have deposited or provided access to graph files (e.g. BED files) for the called peaks.

Data access links
May remain private before publication.

The macroH2A and H2B ChIP-seq data has been deposited in Gene Expression Omnibus, GEO accession number: GSE142082.
https://www.ncbi.nlm.nih.gov/geo/query/acc.cgi?acc=GSE142082

Files in database submission

Raw data files (fastq files) of three replicates and their respective input were deposited including macroH2A1, macroH2A2 and H2B ChIP-seq samples and their processed pileup graph files (TSV files).

Genome browser session
(e.g. UCSC)

All raw and processed data files are available in Geo.

Methodology

Replicates

ChIP-seq samples as well as their respective input samples were derived as biologic replicates from three murine embryonic fibroblast cell lines for each genotype. Each cell line was derived from an individual embryo

Sequencing depth

For ChIP-seq experiment, single-end, 75 bp reads were collected, an average of 65.9 million total reads per sample obtained and an average of 64 million unique reads per sample were present.
Sample_ID Cell description nChIP Total reads Mapped reads Percentage map
KO1_macroH2A1 KO1 MEF macroH2A1 106046394 101704497 95.91%
KO1RS_macroH2A1 KO2 MEF macroH2A1 60311698 58734602 97.39%
KO2RS_macroH2A1 KO3 MEF macroH2A1 64242828 62662046 97.54%
KO6_macroH2A2 KO1 MEF macroH2A2 73396866 72082858 98.21%
KO7_macroH2A2 KO2 MEF macroH2A2 81260704 79708797 98.09%
KO8_macroH2A2 KO3 MEF macroH2A2 65589307 64417740 98.21%
KO1_macroH2A1 KO1 MEF Input 107588112 105289915 97.86%
KO1RS_macroH2A1 KO2 MEF Input 62172490 60879651 97.92%
KO2RS_macroH2A1 KO3 MEF Input 51900612 50816466 97.91%
KO6_macroH2A2 KO1 MEF Input 82684289 81335997 98.37%
KO7_macroH2A2 KO2 MEF Input 73154971 71921345 98.31%
KO8_macroH2A2 KO3 MEF Input 34537094 33994260 98.43%
KO1_H2B KO1 MEF H2B 40194541 38794270 96.52%
KO2_H2B KO2 MEF H2B 46125111 44541659 96.57%
KO3_H2B KO3 MEF H2B 47735443 46046527 96.46%
KO1_H2B KO1 MEF Input 40763729 39875985 97.82%
KO2_H2B KO2 MEF Input 63040158 61773980 97.99%
KO3_H2B KO3 MEF Input 39676607 38839072 97.89%
WT1_macroH2A1 WT1 MEF macroH2A1 139656033 134899826 96.59%
WT1RS_macroH2A1 WT2 MEF macroH2A1 58027460 56900901 98.06%
WT2RS_macroH2A1 WT3 MEF macroH2A1 61056336 59733591 97.83%
WT6_macroH2A2 WT1 MEF macroH2A2 72249028 70314571 97.32%
WT7_macroH2A2 WT2 MEF macroH2A2 69677821 68454534 98.24%
WT8_macroH2A2 WT3 MEF macroH2A2 80782526 79198476 98.04%
WT1_macroH2A1 WT1 MEF Input 137490159 134773802 98.02%
WT1RS_macroH2A1 WT2 MEF Input 59726894 58669919 98.23%
WT2RS_macroH2A1 WT3 MEF Input 60112986 59084083 98.29%
WT6_macroH2A2 WT1 MEF Input 72121925 70901519 98.31%
WT7_macroH2A2 WT2 MEF Input 66689529 65649822 98.44%
WT8_macroH2A2 WT3 MEF Input 81734396 80459689 98.44%
WT1_H2B WT1 MEF H2B 47257584 45665005 96.63%
WT2_H2B WT2 MEF H2B 42959036 41476511 96.55%
WT3_H2B WT3 MEF H2B 39911743 38531071 96.54%
WT1_H2B WT1 MEF Input 42266124 41403609 97.96%
WT2_H2B WT2 MEF Input 47452859 46531099 98.06%
WT3_H2B WT3 MEF Input 53206474 52134315 97.98%

Antibodies

macroH2A1, abcam, Cat.# ab37264;
macroH2A2, abcam, Cat.# ab102126;
H2B, abcam, Cat.# ab1790.

Peak calling parameters

To determine macroH2A peaks, each sample reads were quality trimmed using cutadapt with a minimum quality of 10 and minimum length of 10. Trimmed reads were aligned using bwa-mem with hard clipping on. The bwa-mem output was sorted and indexing using samtools 1.10. The resulting bam files were run through epic2 version 0.0.41 to create peak call in bed files. Peaks were selected with an FDR < 0.01, length > 10Kb, and log2 (Fold Change) > 0.8.

Data quality

RTA 1.8.70.0 software was used for basecall analysis. Reads were trimmed using Cutadapt 1.18 to remove adapters and low-quality flanking regions. These reads were aligned to the mouse genome (MM10) using Bowtie2 2.2.6. with a mismatch error rates ≤ 0.05%. Reads that mapped to multiple locations in the genome were discarded. An average of 98% of total reads per sample were mapped to the genome.

Software

epic2 v. 0.0.41; samtools v. 1.10; macs v. 2.2.6; wigToBigWig v.4; bedops v. 2.4.39; Blat program from UCSC toolkit v. 367; RTA 1.8.70.0 software; Cutadapt 1.18; Bowtie2 2.2.6.

Flow Cytometry

Plots

Confirm that:

- [x] The axis labels state the marker and fluorochrome used (e.g. CD4-FITC).
- [x] The axis scales are clearly visible. Include numbers along axes only for bottom left plot of group (a 'group' is an analysis of identical markers).
- [x] All plots are contour plots with outliers or pseudocolor plots.
- [x] A numerical value for number of cells or percentage (with statistics) is provided.

Methodology

Sample preparation	Cells were detached with trypsin and washed with PBS twice. The cell pellet was resuspended in 500 ul PBS and analyzed within 1 hour.
Instrument	LSR II cytometer (BD Biosciences)
Software	FlowJo
Cell population abundance	The CiA:Oct4 mouse ES cell line is inserted with nuclear GFP reporter gene at the ATG of exon 1 of Oct4, which can express high level of GFP protein. A number of 50,000 cells were assessed for GFP signal each time.
Gating strategy	Cell population of each sample was gated using unmodified mouse ES cells without GFP expression as negative controls. Individual cells were gated based on forward and side scatter, autofluorescent cells were omitted, and remaining cells were analyzed for GFP level.

- [x] Tick this box to confirm that a figure exemplifying the gating strategy is provided in the Supplementary Information.
